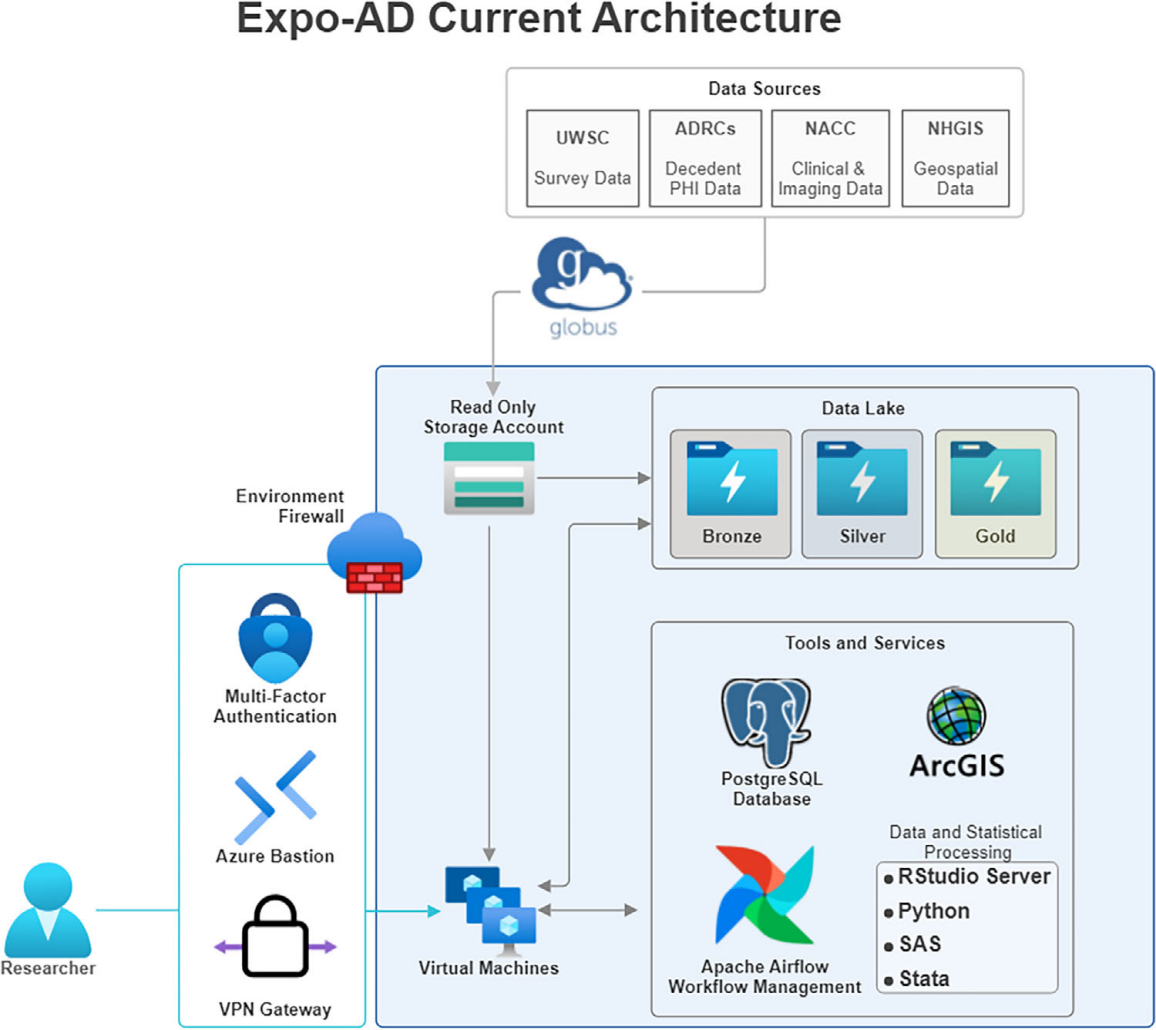# Expo‐AD: An Innovative Alzheimer’s Disease and Related Dementias (ADRD) Research Infrastructure for Secure Data Integration and Exposome Linkage

**DOI:** 10.1002/alz.095640

**Published:** 2025-01-09

**Authors:** Yixuan Cheng, David Lyons, Nyla Thursday, Amanda DeWitt, Barbara B. Bendlin, Amy J.H. Kind

**Affiliations:** ^1^ Center for Health Disparities Research, University of Wisconsin School of Medicine and Public Health, Madison, WI USA; ^2^ Wisconsin Alzheimer’s Disease Research Center, School of Medicine and Public Health, University of Wisconsin‐Madison, Madison, WI USA; ^3^ Department of Medicine, Geriatrics Division, University of Wisconsin School of Medicine and Public Health, Madison, WI USA

## Abstract

**Background:**

Research is needed to understand the impact of the exposome on ADRD, and development of a secure research infrastructure that facilitates linkage of exposome metrics to biological outcomes is critical. Such linkages are challenging because they often require working with protected health information (PHI) covered under the Health Insurance Portability and Accountability Act (HIPAA). In response, we have developed a robust administrative, legal, and cybersecurity infrastructure at the University of Wisconsin (UW) to establish a novel, PHI‐capable, multi‐site service for exposome data linkage for the ADRD research community: “Expo‐AD”.

**Method:**

Expo‐AD is a scalable, cloud‐based environment created and maintained by UW. Data sharing agreements with 22 academic institutions and a specially designed and carefully curated cybersecurity platform provide a secure, PHI‐compliant computing environment.

Expo‐AD utilizes cloud‐based virtual machines (VMs) to ensure isolation of sensitive PHI, maintaining HIPAA compliance and preventing unauthorized access. Data transfer leverages Globus, a research cyberinfrastructure used to reliably and securely share data across institutions. VMs provide a scalable resource which is crucial for managing large datasets and adjusting computational power as needed in exposome research. They also provide disaster recovery capabilities which ensure minimal disruption and safeguard data over time. Access is controlled through both technical and procedural methods and overseen by an external advisory committee

**Results:**

Expo‐AD is currently storing personal identifiers and neuropathology data of 10,875 brain donors acquired from 22 participating brain banks and the National Alzheimer’s Coordinating Center. Residential addresses are geocoded and linked to the Area Deprivation Index to characterize the exposome. Curated data will be released through Globus after each data freeze to requesting researchers. Life course residential history construction for brain donor cases is ongoing.

**Conclusion:**

Expo‐AD is one of the largest multi‐site infrastructures designed specifically to enhance life course exposome intake and linkage to biological ADRD data. Its design facilitates future broad scale sharing of deidentified exposome metrics to non‐PHI capable research resources. Expo‐AD holds the promise of expanding exposome data access across the ADRD field, bringing benefits to the research community and catalyzing collaborative and reproducible exposome research.